# Strategic management accounting and performance implications: a literature review and research agenda

**DOI:** 10.1186/s43093-021-00109-1

**Published:** 2021-11-27

**Authors:** Jafar Ojra, Abdullah Promise Opute, Mohammad Mobarak Alsolmi

**Affiliations:** 1 School of Management, Swansea University, Swansea, UK; 2 GPROM Academic and Management Solutions, Paderborn, Germany; 3 University of Wales Trinity Saint David, Lampeter, UK; 4 Jeddah City, Saudi Arabia

**Keywords:** Strategic management accounting, Competition intensity, Market turbulence, Formalisation, Decentralisation, Organisational strategy, Organisational performance

## Abstract

The important role that management accounting plays in driving organisational performance has been reiterated in the literature. In line with that importance, the call for more effort to enhance knowledge on strategic management accounting has increased over the years. Responding to that call, this study utilised a qualitative approach that involved a systematic review to synthesise existing literature towards understanding the strategic management accounting foundation, contingency factors, and organisational performance impact. Based on the evidence in reviewed literature, we flag key directions for advancing this theoretical premise towards providing further insights that would enable practitioners strategically align their strategic management accounting practices for optimal organisational performance. The limitations of this study have been acknowledged.

## Introduction

Successful managerial decisions enable organisational profitability and accounting aids effective managerial decisions [75]. Aimed at optimising the decision-enabling substance of accounting, management was criticised in 1980s as being too focused on internal operational issues that offer little to management from the point of strategy formulation and sustaining competitive advantage (CIMA Report^1^). Recognising the importance for a broader impact of accounting on managerial decision-making, Simmonds [82, p. 26] introduced and defined strategic management accounting (SMA) as *“the provision and analysis of management accounting data about a business and its competitors, for use in developing and monitoring business strategy”*.

Subsequently there has been increasing efforts that stress the importance for organisations to embrace strategic management accounting theory towards boosting strategic decision-making and organisational performance (e.g. [4, 8, 9, 17, 23, 53, 58, 86, 90, 48], amongst others). As rightly noted by Turner et al. [86], organisations that aim to enhance their competitiveness and performance, must not only develop but also “implement internal policies and procedures such as strategic management accounting that are consistent with their business strategies and account for changing competitive demands” (p. 33). Doing that will enable the strategic management accounting tool to be effectively used to drive corporate success. This is the underlying argument in this study.

The task of profitably satisfying customers is becoming more challenging [61, 65, 67]. Meeting that challenge requires that organisations recognise the importance for effective decision-making. Accountants play a significant role in enabling effective decision-making in organisations (e.g. [21, 23, 27]). Accounting information enables the organisation determine the going concern [6, 36]. Accounting provides the management with relevant information for ensuring and sustaining growth and profitability. The strategic management accounting foundation emphasises that in order to fully fulfil its management decision-making enabling function, accounting practices must not only focus on the internal but also on the external components relating to the organisation's operations. In other words, accounting should embrace a much broader and market-oriented approach and focus on costing (e.g. [8, 17, 58, 78]); planning, control and performance measurement (e.g. [17, 58]), strategic decision-making (e.g. [8, 58]), customer accounting (e.g. [58, 86]) and competitor accounting (e.g. [17, 58, 86]).

Given the importance of strategic management accounting to effective management decision-making and corporate success, there remains a growing interest in understanding the topic. Little wonder therefore that the advocacy for more research towards a better understanding of what strategic management accounting practices organisations adopt and what motivates their preference for one technique over the other (e.g. [4, 53, 58, 86, 90]) remains current. While embracing strategic management accounting is a critical path for enabling effective managerial decision-making and boosting organisational performance (e.g. [3, 9, 58]), the enablement outcome of strategic management accounting practice would hinge on the effectiveness of the organisation in tailoring its strategic management accounting practices to its strategy and environment [9, 11, 58].

Following that contingency logic, this research is a response to the aforementioned call and the aim in this study is to contribute to strategic management accounting discourse by critically analysing the body of knowledge towards enhancing the understanding of how knowledge has evolved in this theoretical domain and also to contribute to knowledge by flagging directions for further knowledge development. To achieve the aim of this study, the theoretical focus in this study is premised along three questions:

1. What strategic management accounting techniques can organisations use towards driving organisational performance?
2. What factors would influence strategic management accounting techniques usage and performance association? and
3. What future research gaps exist based on the explored literature?

## Literature review

### Introduction

This study follows the theoretical foundation that strategic management accounting would aid effective management decision-making, and ultimately boost organisational performance. In line with the aim of this study, relevant literature is reviewed to explain the theoretical premise of this study. The literature review is organised along three core themes in strategic management accounting discourse, namely, strategic management accounting techniques, contingency factors of strategic management accounting usage, and the impact of strategic management accounting on organisational performance.

### Strategic management accounting: definition and techniques

Management accounting is noted to involve the “generation, communication, and use of financial and non-financial information for managerial decision-making and control activities” ([28] p. 3). One major criticism of accounting in the 1980s relates to the fact that accountants have hardly taken a proactive role in the strategic management process [7, 8]. According to Nixon and Burns [55, p. 229], although strategic management has been variously defined, there is “broad consensus that the key activities are (1) development of a grand strategy, purpose or sense of direction, (2) formulation of strategic goals and plans to achieve them, (3) implementation of plans, and (4) monitoring, evaluation and corrective action”. The role of management accounting is to enable effective decision-making, and it involves typically information gathering and analysis, identifying options, implementation, monitoring and evaluation [16]. Thus, the focus in strategic management accounting, rephrased also as accounting for strategic positioning [73, 74], is to embrace a broader approach that incorporates a strategic management focus into its dynamics towards effectively enabling management decision-making and organisational performance [8, 80]).

Since the first attempt by Simmonds [82, p. 26] who defined strategic management accounting as *“the provision and analysis of management accounting data about a business and its competitors, for use in developing and monitoring business strategy”*, there have been numerous attempts to enhance that definition and identify core techniques of strategic management accounting. For example, CIMA [16] describes strategic management accounting as a management accounting form that emphasises focusing on information relating to external factor of the entity and also on non-financial information as well as information that is generated internally. In a much earlier contribution, Bromwich [7, p. 28] offers a description of strategic management accounting as involving “the provision and analysis of financial information on the organisation’s product markets and competitors’ costs and cost structures and the monitoring of the organisation’s strategies and those of its competitors in the market over a number of periods” (Cited in [56, p. 14]).

In their 2008 study, Cadez and Guilding asked the question “what is strategic management accounting?” (p. 838). In that same study, they conclude, based on evidence from reviewed literature, that there are two perspectives of strategic management accounting. While one perspective focuses on strategically oriented accounting techniques, the other focuses on the actual involvement of accountants in the strategic decision-making process. Following the former perspective (e.g. [8, 9, 17, 58]), existing literature distils sixteen (16) strategic management accounting techniques that are categorised under five SMA themes (e.g. [9, 11, 58]):

1. Strategic costing;
2. Strategic planning, control and performance measurement;
3. Strategic decision-making;
4. Competitor accounting; and
5. Customer accounting.

#### Strategic costing

According to literature (e.g. [8, 11, 23]), strategic and marketing information-based cost data can be leveraged by organisations to ensure effective strategies for achieving sustainable competitive advantage. Thus, organisations must recognise the importance of integrating cost strategies and undertake multiple strategic cost analyses. Literature distils five key costing techniques: attribute costing (e.g. Roslender and Hart 2003), life-cycle costing (e.g. [8, 17]), quality costing (e.g. [17]), target costing (e.g. [8, 17]) and value chain costing (e.g. [8]).

#### Strategic planning, control and performance measurement

Literature has also underlined the need for organisations to give due attention to planning, control and performance measurement features of the strategic management accounting, as doing that is important in the pro-active market orientation approach for competing effectively in the marketplace (e.g. [8, 58], Chenhall 2005). Core components under the strategic planning, control and performance measurement tool includes benchmarking (e.g. [8, 17]) and integrated performance management (Balanced Scorecard) (e.g. [8, 17]).

#### Strategic decision-making

As a strategic management accounting tool, strategic decision-making is a critical tool for supporting strategic choice [11]. Core strategic decision-making options include strategic costing (e.g. [58]), strategic pricing (e.g. [11, 58]) and brand valuation (e.g. [11, 58]).

The importance of addressing strategic costing as a key strategic decision-making element has been emphasised in the literature (e.g. [58, 78, 79]). In this discourse, it is underlined that effectively driving competitive advantage requires cost analysis that explicitly considers strategic issues. In line with that viewpoint, Cadez and Guilding [8] note that strategic costing involves “the use of cost data based on strategic and marketing information to develop and identify superior strategies that will produce a sustainable competitive advantage” (p. 27).

In the literature too, strategic pricing is underlined as another core element the strategic decision-making typology of strategic management accounting (e.g. [8, 58], Simmonds 1982). According to scholars, understanding market competition level, which as noted by Guilding et al. [29, p. 120] entails the appraisal of the following factors: “competitor price reaction, price elasticity; projected market growth; and economies of scale and experience”, is important (e.g. [8, 11, 58]).

Within the strategic management accounting literature, brand valuation is the third element of the strategic decision-making technique. The brand valuation component “involves combining projected brand earnings (an accounting-orientated measure) with a multiple derived from the brand’s strength on strategic factors such as the nature of the brand’s market, its position in that market and its level of marketing support” [29, p. 118]. In the view of Cescon et al. [11], brand valuation enables organisations to understand market reputation trends over time and potential implications for marketing executives and strategic accounting. Cescon et al. [11] contend that organisations would achieve a variable brand valuation that would provide a potential measure of marketing achievement when perceived quality and branded products are considered, while Guilding et al. [29] remind that achievable impact of brand valuation would hinge, amongst others, on the valuation method used.

#### Competitor accounting

According to Porter [72], strategy involves developing appropriate tools that enable a firm to analyse and determine its position in a competitive market. Thus, a firm selects suitable strategies that enables it compete more effectively over its rivals. To effectively do that, a firm needs to collect competitor accounting information. The importance of giving due attention to competitor accounting has been underlined in the literature (e.g. [11, 17, 58]). Three forms of competitor accounting tools are described in the literature, namely, competitor cost assessment (e.g. [11, 17, 58]), competitor position monitoring (e.g. [11, 58]) and competitor performance appraisal (e.g. [11, 17, 58]).

#### Customer accounting

The fifth cluster of strategic management accounting techniques described in the literature relates to customer accounting (e.g. [49, 58]). Customer accounting concerns practices aimed at appraising profit, sales or costs related to customers or customer segments [58]. Core customer accounting techniques include customer profitability analysis (e.g. [30, 58]), lifetime customer profitability analysis (e.g. [58]) and valuation of customers as assets (e.g. [30, 58]).

### The contingency factors of strategic management accounting

#### Introduction

According to management accounting discourse, when organisations carefully embrace appropriate strategic management accounting practices, they would ensure successful managerial decisions that would ultimately lead to optimising organisational performance (e.g. [48, 53, 56, 58]). Thus, the extent of improved performance that an organisation would achieve would depend on its careful utilisation of appropriate strategic management techniques. As noted by Roslender and Hart (2003), p. 4 and further supported by subsequent literature (e.g. [34, 58]), “the adoption of strategically oriented management accounting techniques and accountants’ participation in strategic management processes”, is a core research premise. In line with the carefulness notion mentioned above, the contingency perspective has been widely utilised in the effort to understand strategic management accounting practices and performance impact (e.g. [8, 12, 30, 34, 58]). The underlying foundation in the contingency perspective is based on the notion “that an organisation maximises its efficiency by matching between structure and environment” [22, p. 49]. According to Otley [68]:

The contingency approach to management is based on the premise that there is no universally appropriate accounting system that applies equally to all organisations in all circumstances. Rather, it is suggested that particular features of an appropriate accounting system will depend on the specific circumstances in which an organisation finds itself. Thus, a contingency theory must identify specific aspects of an accounting system which are associated with certain defined circumstances and demonstrate an appropriate matching (p. 413).

Thus, the central foundation in the contingency perspective is that no one single accounting system is universally fit for all organisation in all circumstances (e.g. [41]). No one accounting control system can be seen as “best” for all situations; rather, the appropriateness of any control system would depend on the organisation's ability to adapt effectively to the environment surrounding its operations [41, 58, 86].

From reviewed literature, numerous researchers have flagged key contingency factors that should be considered in relation to strategic management accounting practice. Four factors were identified as critical contingency factors in the strategic management accounting systems design in Cadez and Guilding's [8] study, namely: business strategy, strategy formulation pattern, market orientation and firm size. On their part, Islam and Hu [41] identify core organisational effectiveness factors to include technology, environmental volatility, organisational structure, information system and size of the organisation.

Analysed together, the conceptualisation in the aforementioned studies [8, 41] reflect perspectives that have been recognised in the 1980s. For example, Merchant [50] describe contingency factors to include firm size, product diversity, extent of decentralisation and budgetary information use. In their study of accounting information systems, Gordon and Narayanan [26] classify three core contingency factors to include perceived environmental uncertainty, information characteristics and organisational structure. Based on a study that examined the extent to which accountants were involved in the strategic management process, CIMA^2^ reports three key contingency factors: “organisational influences, accountant led influences and practicalities” (p. 12). Exploring strategic management accounting practices in the Palestinian context, Ojra [58] conceptualised a comprehensive contingency perspective that considered (1) organisational structure (involving formalisation and decentralisation), (2) organisational size, (3) technology and (4) organisational strategy. In more recent literature, Pavlatos [70] suggests seven factors that affect strategic management accounting usage in the hospitality industry (hotels) in Greece, namely, “perceived environmental uncertainty, structure, quality of information systems, organisational life cycle stage, historical performance, strategy and size” (p. 756).

The contingency framing in this study draws from the theoretical guideline which suggests that both the internal and external environments of organisations should be considered in the effort to advance strategic management accounting literature (e.g. [58, 70]). The conceptual framing in this study includes two external (perceived environmental uncertainty—competitive intensity, and market turbulence) and three internal (organisational structure—formalisation, and decentralisation, and organisational strategy) factors.

#### Perceived environmental uncertainty and strategic management accounting usage

From the perceptual lens, the environment could be viewed as certain or uncertain only to the extent that decision makers perceive it to be (e.g. [1, 11]). Perceived environmental uncertainty is described as the absence of information relating to organisations, activities and happenings in the environment [20]. According to Cescon et al. [11], organisations must give due attention to their operational environment because engaging with environmental uncertainty factors would enable them identify key change drivers.

Prior literature has documented that perceived uncertainty significantly influences the extent to which firms would embrace strategic management accounting practices (e.g. [49, 58, 70]). According to that foundation, how firms respond from the point of strategic management accounting practices that they would endorse would depend on the nature of environmental uncertainties that surround their operational activities.

Studying the hotel property setting, Pavlatos [70] documents a positive correlation between the degree of environmental uncertainty and the use of strategic management accounting tools. In other words, the higher the perceived environmental uncertainty, the higher the need for use of strategic management accounting tools. Intensified use of strategic management accounting tools is essential because that will enable the hotels to manage the uncertainties, and be more effective in managerial decision-making, and ultimately improves organisational performance [70]. The notion of a significant influence of environmental uncertainty on strategic management accounting practices is supported by prior literature (e.g. [15]). According to them, managers who operate in highly uncertain environments would require information that is timely, current and frequent. Other scholars have also argued that environmental uncertainty would be associated with more pro-active and externally focused accounting systems (e.g. [32, 38]).

In their study of Italian manufacturing companies, Cescon et al. [11] found a positive association of perceived environmental uncertainty and strategic pricing usage as a feature of the strategic decision-making SMA technique. In other words, the more the perceived environmental uncertainty, the higher the usage of the strategic pricing feature of the strategic decision-making SMA component.

In the perceived environmental uncertainty literature, two core dimensions have been distilled, namely competitive intensity and market turbulence (e.g. [30, 58]). Market turbulence—a subset of environmental turbulence [47], is defined by Calantone et al. [10] as characterised by continuous changes in customers’ preference/demands, in price/cost structures and in the composition of competitors. In settings where there is high market turbulence, organizations would need to modify their products and approaches to the market more frequently [44]. On the other hand, the notion of competitive intensity relates to the logic that organisations compete for numerous resources, such as raw materials, selling and distribution channels, as well as quality, variety and price of products [26, 46]. Achieving organisation-environment alignment in highly competitive environments requires that organisations have the capacity to effectively detect environmental signals and timely communicate environmental information (e.g. [88]).

Exploring Australian hospitality industry, McManus [49] examined the association of competition intensity and perceived environmental uncertainty on customer accounting techniques usage. The study suggests that competition intensity positively associates with customer accounting practices but also found that higher perceived environmental uncertainty would not lead to greater usage of customer accounting techniques in the explored hotels. In a much similar conceptualisation, Cescon et al. [11] examined the association of environmental uncertainty and competitive forces on strategic management accounting techniques usage in large Italian manufacturing firms. Empirically, that study found that external factors (environmental uncertainty and competitive forces) positively associate with SMA usage (strategic pricing, balanced scorecard, risk analysis, target costing, life-cycle costing). Based on the two-dimensional conceptualisation, Ojra [58] examined the relationship between perceived environmental uncertainty and SMA usage in Palestinian firms. Ojra [58] hypothesised a positive correlation of perceived environmental uncertainty (conceptualised to include competition intensity and market turbulence) but found no support. To the contrary, Ojra [58] documents a potential for negative influence of perceived environmental uncertainty on strategic management accounting techniques usage, however only significant for the market turbulence dimension. In other words, Ojra [58] suggests that market turbulence associates negatively with strategic management accounting techniques usage in medium Palestine firms.

#### Organisational structure (formalisation) and strategic management accounting usage

Across the various streams of management, formalisation has been mentioned as a key contingency factor in understanding the operational dynamics of organisations (e.g. [58, 63, 64]). With regard to strategic management accounting discourse, this notion has been numerously supported (e.g. [26, 58, 85]).

Studying the influence of formalisation in the functional relationship between the accounting and marketing departments, Opute et al. [64] suggest a positive association. In other words, they argue that the more formalised the processes in the firm, the higher the achieved integration between both functional areas. However, Opute et al. [64] note that whether this positive association is achieved would depend on the integration component (information sharing, unified effort and involvement) considered.

In the strategic management accounting domain, there is mixed evidence of the association of organisational structure on strategic management accounting usage. For example, Ojra [58] hypothesised that less formalised organisational structure would lead to higher use of strategic management accounting techniques in Palestinian firms but found no support for that hypothesis. In that study, no support was found for the notion that less formalised structures would lead to higher use of strategic management accounting techniques, both for total SMA as well as for all the dimensions of SMA. Thus, that study concludes that formalisation has no significant influence on strategic management accounting techniques usage in Palestinian firms. That conclusion supports the findings in Gordon and Narayanan [26], but contrast the view in Tuan Mat’s [85] exploration of management accounting practices.

#### Organisational structure (decentralisation) and strategic management accounting usage

Similar to formalisation, management scholars have noted decentralisation as a core organisational structure factor that should be given due attention in the drive to enhance the understanding of contingency theory (e.g. [58, 62, 63]). Organisational structure has been noted to influence the strategic management accounting practices of a firm (e.g. [58, 70]). Within that foundation, decentralisation (or its opposite) has been flagged as a major factor. A contention that has been underlined numerously in the discourse is that strategic management accounting usage would be higher in organisations that embrace decentralised structure. Following that foundation, Pavlatos [70] hypothesised that SMA usage is higher in decentralised hotels than in centralised hotels in Greece. The results support the hypothesis: there is higher need for strategic management accounting practices in decentralised firms, as lower-level managers require more information to aid decision-making.

The above conclusion supports as well as contrasts prior literature, namely Chenhall [14] and Verbeeten [87], respectively. According to Chenhall [14, p. 525], “strategic management accounting has characteristics related to aspects of horizontal organisation as they aim to connect strategy to the value chain and link activities across the organisation…”. Chenhall [14] adds that a typical approach in horizontal organisation is identifying customer-oriented strategic priorities and then exploiting process efficiency, continuous improvements, flattened structures and team empowerment, to initiate change, a conclusion that suggests that higher use of strategic management accounting practices would seem ideal in such decentralised organisational structure. The reason for that outcome is that in decentralised structure, lower-level managers can adapt their MACS as necessary to meet requirements [52], a logic that finds support in McManus [49] who found that customer accounting usage is higher in Australian hotels that are decentralised than those that are centralised. In contrast to that logic, Verbeeten [87] found decentralisation to associate negatively with major changes in the decision-influencing components of MACS.

Insight about the less developed context, namely about Palestinian firms lend support to, as well as contrast past literature. According to Ojra [58], decentralisation has a tendency to associate negatively with strategic management accounting usage. Therefore, although statistically insignificant, the results indicate that explored Palestinian firms that endorse decentralised decision-making process would seemingly have lesser need for strategic management accounting practices. On the evidence that decentralisation may have a negative influence on strategic management accounting usage, Ojra [58] supports Verbeeten [87] but contrasts Pavlatos [70].

#### Organisational strategy and strategic management accounting usage

An internal organisational factor that has been considered important in the understanding of contingency perspective of management accounting relates to organisational strategy (e.g. [8, 17, 58]). Hambrick [33] defined strategy as:

A pattern of important decisions that guides the organisation in its relationship with its environment; affects the internal structure and processes of the organisation; and centrally affects the organisation’s performance (p.567).

In the strategic management accounting discourse, organisational strategy has been mentioned as one of the key factors that would condition strategic management accounting practices of a firm (e.g. [9, 58, 70, 86]). For example, Turner et al. [86] note that in hotel property setting, strategic management accounting use would hinge on the market orientation business strategy of the firm. Given the notion that strategic management accounting would aid management decision-making and lead ultimately to improved organisational performance, there is some legitimacy in expecting that organisations that align their strategic management accounting practices to the strategic orientation of the firm would achieve a higher organisational performance.

Following Miles and Snow’s [51] strategy typology (prospector, defender, analyser, and reactor), efforts to understand the association of strategy to strategic management accounting tools usage have also tried to understand how the various strategy typologies play out in this association. For example, Cadez and Guilding [9] considered the prospector, defender and analyser typologies in the Slovenian context, while Ojra [58] considered the prospector and defender typologies in the Palestinian contexts.

Cadez and Guilding [9] report that companies that endorse the analyser strategy, which is a deliberate strategy formulation approach, are not highly market oriented, but tend to show high usage of SMA techniques, except for competitor accounting technique. Further, they report that prospector strategy-oriented companies also pursue a deliberate strategy formulation approach, but are highly market oriented, and SMA techniques usage is fairly high (for competitor accounting) and averagely high (for strategic costing). For very high prospector-oriented companies, they are highly market oriented, have a strong strategy drive and a very high SMA techniques usage. For the defender strategy-type companies, they suggest that such companies are not only average in their market orientation, but also in their usage of SMA techniques.

In the study of Palestinian companies, Ojra [58] offers insights that resonate relatively with the findings in Cadez and Guilding [9]. Ojra [58] suggests that prospector companies have a higher usage of SMA techniques than defender-type companies. So, SMA technique usage is positively associated to prospector strategy (see also [8]. Elaborating the findings, Ojra [58] reports that prospector-type companies focused more on four SMA techniques (mean values reported), namely SMAU-Planning, Control and Performance Measurement (4.601), SMAU-Strategic Decision Making (4.712), SMAU-Competitor Accounting (4.689) and SMAU-Customer Accounting (4.734), statistical results that are significantly higher than the results for 'defender'-type companies. Cinquini and Tenucci [17] lend support to Ojra [58]: 'defender'-type companies give more attention to the Costing dimension of SMA.

Without emphasising the strategy typologies, Pavlakos (2015) comments that organisational strategy affects SMA usage in the Greek hotel industry.

### Strategic management accounting and organisational performance

A central tenet in the strategic management accounting foundation is that management accounting would significantly aid organisations to achieve sustained competitiveness [7, 82]. Implicitly, these scholars argue that in order to stay competitive in the marketplace, organisations should not only focus on cost-volume-profit issues, but rather embrace a broad externally focused management accounting approach that is strategically driven and provides financial information that enables management to effectively formulate and monitor the organisation's strategy. Thus, management accounting should also focus on competitor information as that will enable management effectively organise the firm's strategic structure.

Over the years, there is growing recognition of the importance of strategic management accounting to organisations, leading therefore to increasing research attention. One area that has received attention in the strategic management accounting discourse relates to the organisational performance enhancement notion (e.g. [23, 56, 58, 77, 86]).

Insights from Malaysia also add to the discourse on the impact of strategic management accounting usage on organisational performance. In their study of Malaysian electrical and electronic firms, Noordin et al. [56] examined the extent of usage of strategic management accounting and influence on the performance of the participating firms. The study found that in explored Malaysian companies, the extent of strategic management accounting usage was significantly related to organisation’s performance. That conclusion supports Cadez and Guilding [8] who contend that there is a positive association between strategic management accounting usage and organisational performance.

In a performance perspective that considers the ISO 9000 Quality Management System (QMS) aspect, Sedevich-Fons [77] examined the connection between strategic management accounting and quality management systems performance. The findings show that strategic management accounting and quality management are complementary and their effective implementation would enhance overall performance. Sedevich-Fons [77] notes further that when both are used in conjunction that would spread SMA techniques and enable full exploitation of Quality Management Systems.

Insights from the less developed economy context also associate organisational performance to the implementation of strategic management accounting practices (e.g. [3, 57, 58]). In a conceptual approach that aimed to address one major gap in previous literature, Ojra [58] examined both the financial and non-financial dimensions of organisational performance. According to Ojra [58], strategic management accounting usage does not impact the financial dimension of organisational performance but exerts significant positive impact on non-financial performance. That finding resonates with Perera et al. [71] conclusion that various forms of management accounting associate positively with the use of non-financial measures.

On their part, Oboh and Ajibolade [57], in their investigation of the association between strategic management accounting practices and strategic decision-making in Nigerian banks, found that explored Nigerian banks “practice SMA not as a concept, but as a principle of operation, and that SMA contributes significantly to strategic decision-making in the area of competitive advantage and increased market share” (p.119).

Alabdullah [3] offers evidence that adds support to the insights in the aforementioned studies [57, 58]. In a study that explored the Jordanian service sector, Alabdullah [3] found that strategic management accounting enables performance in the service sector in Jordan. If strategic management accounting is effectively implemented, that will enable optimal strategic decision-making and ultimately improve organisational performance.

## Research methodology

### Research design

Qualitative research method [18, 76] is used in this study to achieve the objectives of this research. Following the methodological approach, as well as responding to the research call, in a past study on the contingency perspective of strategic management accounting [41], a study which was literature review-based, literature review-based qualitative research approach was deemed fit in this study.

A systematic review approach (e.g. [5, 39, 81]) is used in this research on the topic of strategic management accounting. Using the systematic review approach in this study is appropriate because it enables a systematic and transparent approach in identifying, selecting, and evaluating relevant published literature on a specific topic or question [42, 83]. Furthermore, systematic review approach was deemed appropriate for this study as it has been documented to aid core research gaps identification and steering future research (e.g. [40, 59, 66]).

Alves et al. [5] forward a two-stage guideline for systematic review of literature: planning the review and conducting the review and analysis. As they noted, researchers should describe how the systematic approach was planned (in the former) and also describe the phases of the review and selection of literature (in the latter). In this research, effort was made to combine the best evidence: careful planning was used to determine literatures for inclusion or exclusion (e.g. [5, 65, 67]). The planning was focused at identifying relevant publications in various academic journals on the topic of strategic management accounting. First, the theoretical themes to be considered in the conceptual premise of this study were confirmed and academic resource for tracking relevant publications determined [5, 66, 83].

In the preliminary stage of the literature review, electronic search was carried out to identify relevant literature relating to strategic management accounting. Three steps were taken in the systematic review: we searched the literature, analysed and synthesised the literature, and wrote the review. Several databases were scanned using key search terms to capture relevant literature [81]. Core search terms were used, such as strategic management accounting, historical aspects of strategic management accounting, contingencies of strategic management accounting practices, strategic management accounting and organisational strategy, strategic management accounting and organisational performance, amongst others. Relevant publications were also found using data extraction tools such as Google Scholar, Emerald Insight and Research Gate.

Using the aforementioned methodological approach, a collection of relevant articles published in academic journals was identified. Identifying the relevant literature in this study followed methodological guideline [69]: criteria of language, relevance and type of research to identify relevant studies were embraced, and articles that contained non-English contents and also articles that did not fit closely to the thematic premise of this study were excluded. It is important to emphasise here that this study recognises that not all publications relating to the topic of strategic management accounting may have been considered in this research. However, for the scope of this piece of research, the body of literature covered in this study was deemed adequate for the conceptual framing.

Table 1 shows a sample of selected literature covered in this piece of research, pinpointing clearly the focus, context of the studies and findings from the studies.

**Table 1:** A sample of selected studies reviewed in this research

S/Nr	Studies	Theoretical focus and geographical setting	Core arguments (or facts)
1	Cadez and Guilding [9]	Examined the effectiveness of various types of strategy and SMA and also how horizontal and vertical alignment of management accounting and strategy facilitate Slovenian firms' performance	There are various levels of performance and each depends on the degrees of fit. Different strategic and structural alternatives influence similar levels of performance. Internally consistent configurations lead to higher performance
2	Noordin et al. [56]	This study examined explored strategic management accounting usage and performance impact in Malaysian electrical and electronic companies	The study found that in explored Malaysian companies, the extent of strategic management accounting usage was significantly related to the organisation’s performance
3	Ojra [58]	Using a comprehensive contingency framework, this study examined the strategic management accounting nature and performance implications in Palestinian firms	The study suggests that perceived environmental uncertainty would lead to decentralised organisational structure, lead to increase in the use of non-financial performance, and also that perceived environmental uncertainty would be greater in prospector-strategy than defender-strategy Palestinian firms. The study however found no support that higher environmental uncertainty would lead to higher SMA usage. The study also found no support for the hypothesis that organisational structure (less formalisation and more decentralisation) would lead to higher SMA usage. The study notes that there is higher SMA techniques usage in prospector than defender-type companies. On the point of performance, the study found that greater SMA usage is positively associated to non-financial performance but not to financial performance
4	Turner et al. [86]	This study explored hotel property performance and strategic management accounting influence	The study suggests that hotel property SMA usage would depend on market orientation business strategy. Further, the study contends that hotel property SMA usage as well as hotel property customer performance impact mediating influence on how hotel property market orientation business strategy use associates with hotel property financial performance
5	Zamecnik and Rajnoha [90]	The study explored strategic business performance management in Slovak enterprise industry	The study found that in understanding strategic business performance, the central focus should be on endorsing a comprehensive strategic orientation rather focusing mainly on the financial aspect
6	Henri et al. [34]	The study examined the relationship between executional cost management and structural cost management components of SCM, financial performance, in Canadian manufacturing firms	There is a direct and positive effect of tracking environmental cost on financial performance. Also, implementation of environmental initiatives associates positively with financial performance. Indeed, both executional cost management (the tracking of environmental costs) and structural cost management (the implementation of environmental initiatives) enhance financial performance
7	Alabdullah [3]	The study examined the impact of strategic management accounting on performance in the Jordanian service sector	The study suggests that strategic management accounting enables performance in the service sector in Jordan. If strategic management accounting is effectively implemented, that will enable optimal strategic decision-making and ultimately improve organisational performance
8	McManus [49]	This study explored the contingency perspective of strategic management practices in the Australian hotel industry	The study suggests that Australian hotels that are highly market orientated and prioritise a decentralised structure use more customer focused accounting and marketing practices. Also, the study suggests a significant positive association between market orientation and a prospector-type strategy, and also between market orientation and both financial and non-financial performance
9	Cescon et al. [11]	This study examined the association between strategic choices and use of strategic management accounting techniques in large Italian manufacturing firms	This study contends that strategic management accounting usage is not dependent on strategy type, but however marginally dependent on geographic orientation.
10	Sedevich-Fons [77]	This study explored strategic management accounting and quality management performance impact	The findings show that strategic management accounting and quality management are complementary and their effective implementation would enhance overall performance. When used in conjunction, that would spread SMA techniques and enable full exploitation of Quality Management Systems
11	Pavlatos [70]	This study examined the factors that influence strategic management accounting usage in the hotel industry in Greece	The study concludes that seven factors that affect strategic management accounting usage in the hospitality industry (hotels), namely, “perceived environmental uncertainty, structure, quality of information systems, organisational life cycle stage, historical performance, strategy and size” (p.756)
12	Oboh and Ajibolade [57]	This study examined strategic management accounting practices and strategic decision-making impact in Nigerian banks	This study found that explored Nigerian banks “practice SMA not as a concept, but as a principle of operation, and that SMA contributes significantly to strategic decision-making in the area of competitive advantage and increased market share” (p.119)
13	Alamri [4]	This study examined the association between strategic management accounting facets and organisational performance in Saudi firms	The study contends that there is a significant positive association between strategic management accounting facets and organisational performance, both financial and non-financial components
14	Cinquini and Tenucci [17]	This study explored the influence of business strategy (3-components) on strategic management accounting techniques usage in Italian manufacturing firms	The study found that defender-type firms have greater use of SMA costing techniques while for prospector-type firms, no relationship was found between strategic pattern and SMA orientation. For the mission variable, the study shows that build companies are more willing than harvest companies to use SMA customer oriented techniques
15	Cadez and Guilding [8]	The study explored strategic choices, market orientation, and company size on two dimensions of strategic management accounting (SMA), and mediating influence of SMA on performance in Slovenia	The study found a significant positive association of prospector strategy, deliberate strategy and company size on SMA usage, as well as a significant impact of SMA usage on performance. Also, the study found that market orientation does not associate with SMA usage but associates strongly with performance
16	Hoque [37]	The study explored the role of performance measures choice on the relationship between (a) strategic priorities and performance and (b) environmental uncertainty and performance	The study suggests a significant positive association between management’s strategic choice and performance when non-financial performance measures are used. On the other hand, no significant relationship between environmental uncertainty and use of non-financial performance measures was found
17	Mohammed et al. [53]	The study explored the impact of strategic management accounting information on organisational performance in Malaysian hospitals	The study suggests that strategic management accounting information (which included competitor, client and product data) impacts positively on the performance of explored private Malaysian hospitals
18	Chenhall [13]	This study reviewed the findings in prior literature ( over 25 years up to the time of the study) on the contingencies of management control systems (MCS)	This critical review examined previous literature on how the nature of environment, structure, size, technology, strategy and national culture explain the effectiveness of management control systems. The review explored MCS purpose, core elements, as well as the meaning and measurement of contextual variables
19	Cuganesan and Dunford [19]	This study examined strategic management accounting and strategy practices in the public sector	The study identifies critical management accounting roles in strategising beyond the typically ascribed functions of decision-facilitation and decision influencing. The study indicates that public sector entities are using particular management accounting techniques for strategising, which offers a counter-point to private sector insights that till date has dominated SMA research
20	Jusoh [43]	The study explored the influence of perceived environmental, firm size uncertainty, and business strategy on the use of multiple performance measures in Malaysian firms	The study found that perceived environmental uncertainty negatively influences the extent financial and internal processes measures are used, while prospector strategy exerts a positive influence on the use of innovation and learning and overall balanced scorecard (BSC) measures. Also, the study contends that analyzer strategy associates positively with the use of time-focused customer measures, while firm size positively impacts the use of innovation and learning measures
21	El Deeb [23]	This study explored strategic management accounting and performance impact in healthcare industry in Cairo, Egypt	Appropriate cost management practices will enable organisations to effectively monitor the external environment towards appropriate and effective strategies. Utilising appropriate costing system (full absorption costing, marginal costing, activity-based costing and standard costing will enable proper identification of costs and profitability of hospital units
22	Acquaah [2]	This study explored relationships between management control systems (MCS), business strategy and firm performance in family businesses (FBs) and non-family businesses (NFBs) in Ghana	The study found that the influence of (i) diagnostics control systems (DCS) on cost leadership strategy is higher for NFBs than FBs; (ii) interactive control systems (ICS) on differentiation strategy is higher for FBs than NFBs. Also, the study contends that the impact of the dynamic tension created by concurrently using DCS and ICS on both the cost leadership and differentiation strategies is higher for FBs than NFBs. Furthermore, it contends that business strategy mediates the association between MCS and performance, but the impact of MCS on performance (both indirect and total) are stronger for FBs than NFBs

Content analysis in this study followed the inductive lens [84]. This involved thorough reading of the selected studies to capture the state of knowledge in the domain of this study (e.g. [65]). To ensure that knowledge is captured accurately, an iterative approach that involved reading the text back and forth was used (e.g. [65]). Analysing the relevant literature, we focused on identifying the core thematic threads in the discourse (see Table 1) in each selected paper. In concordance with the central aim of this review, data abstracted were in the form, effect and findings, and conceptualisation of ideas or theoretical perspectives [83]

### The analysis

The interpretive approach of analysis was followed in processing the qualitative data to achieve reliable meaning in this study (e.g. [59, 65, 67, 84]). Following that precedence, an iterative approach that involved reading reviewed literature back and forth, was used in this study. Using that approach, a synthesis of literature was undertaken to capture the core threads, debates and themes in the literature (e.g. [65, 67]). Guided also by that methodological approach, relevant directions for future research have been flagged towards enhancing the knowledge about strategic management accounting and performance association.

Subjectivity is a major concern in qualitative researches (e.g. [18, 76]). To address that concern, steps taken in this research to validate the articles incorporated into this research include rigor of conduct and strength of evidence by cross-referencing, as well as undertaking a duplicate check (e.g. [76, 81]).

## The findings

Prompted by the central threads that emerged from the analysis of the selected literature, the findings from this study are organised along three core themes: strategic management accounting techniques, contingencies of strategic management accounting techniques usage and the organisational performance implications of strategic management accounting usage.

The importance of management accounting, and in particular the strategic management accounting element as a tool for enabling top management to make effective decisions that enable organisation compete effectively in the marketplace, is gaining increasing mention in management discourse. In that discourse, five core categorisations of SMA techniques: strategic costing; strategic planning, control and performance measurement; strategic decision-making; competitor accounting; and customer accounting. While literature distils numerous forms of strategic management accounting techniques that organisations may embrace towards enabling effective management decision-making and organisational performance, evidence was found in reviewed literature that in some organisations, practitioners do not believe that strategic management accounting as a separate concept is a notion they subscribe to (e.g. CIMA^3^; [48, 55]). For example, CIMA^4^ documents that participants unanimously do not subscribe to the notion, a conclusion which lends support to prior literature [48, 55] that notes that strategic management accounting as a term, did not exist in the lexicon of accounting practitioners.

Grounded on the substance that effective use of the SMA techniques would improve organisational performance, immense research effort has focused on how organisations can effectively align the SMA usage towards achieving desired performance improvement. Premised in that theoretical domain, this study examined existing literature on the contingency factors of competitive intensity, market turbulence, formalisation, decentralisation and organisational strategy and SMA usage. Cumulative evidence obtained from the review of literature reinforces the need for organisations to pay particular attention to their operational environment in their use of SMA techniques. Reinforcing the fit principle, the cummulative evidence underlines that optimising the benefits of the strategic management accounting techniques in enabling effective customer orientation and boosting organisational performance is dependent on the organisation's ability to effectively align strategic management accounting practices to its operational environment. In other words, what works for an organisation would depend on the organisational dynamics, internal, as well as external. For example, formalisation may work for some but not for some, as decentralisation could work for some but not for some. Similarly, the utility of SMA techniques would hinge on the competitive intensity and market turbulence features of an organisation. Thus, aligning SMA practices to the internal and external features of an organisation is essential to enable them adapt effectively to their circumstances, make rational decisions and optimise their performance. So, alignment is critical because there is no one-size fits all approach for achieving customer orientation and organisational performance goals.

The third focal point of this study relates to the association of SMA techniques usage to organisational performance. Reviewed literature shows that organisations are achieving higher performance through the use of SMA techniques. In other words, effective use of SMA techniques would improve organisational performance. The plausibility in that performance outcome lies in the fact that organisations are able to utilise appropriate SMA measures to ensure effective, customer, competitor, strategic decision-making, costing, and planning and control orientation in their operational activities. Further on the performance point, literature also suggests that management control systems (MCS)–performance relationship is mediated by business strategy (e.g. [2]). Also, that study documents that the impacts (both indirect and total) of MCS on performance are stronger for family businesses than non-family businesses.

## Conclusions

### Conclusions and implications

One of the major challenges that organisations are facing in today's dynamic marketplace is to steer their organisations in a way that they can stay competitive. In the contemporary world, where globalisation and technological evolution have expanded the options that customers have (e.g. [31, 61, 65, 67]), organisations must strive hard to win the loyalty of customers. For organisations wishing to achieve that, strategic management accounting practices offer a strategic pathway. Organisations must embrace strategic management accounting practices that would enable them understand the market, their competitors, and the customers and leverage the intelligence from that knowledge to organise their operations towards profitably satisfying the customer. To effectively do that, organisations must avoid the mistake of focusing only on the internal issues; rather, their effort must be tailored towards embracing strategic management accounting practices that would enable them to be fully informed of the market trends, customer dynamics and competitor trends. Thus, organisations must ensure that good costing, planning, control and performance measurement; strategic decision-making, customer accounting and competitor accounting measures are embraced to enable them compete effectively.

Furthermore, in that drive to compete effectively in the market and profitably satisfy customers, organisations would not only need to embrace appropriate strategic management accounting techniques but also do that bearing in mind the environments that surround the operational activities. In other words, organisations must give due attention to the contingencies of their operational setting. Organisations must ensure a good blend of critical factors that would enable their optimal operation. Due attention must be given to organisational structure (centralisation or decentralisation of decision-making process), external environment (dynamism and turbulence), technological development, strategic approach, size of the organisation, amongst others. Doing that is critical for corporate success because there is no one size fits all approach—the outcome achieved would depend significantly on the dynamics surrounding the operational activities of the firm.

Thirty-three months on after Covid 19 was documented,^5^ the pandemic is still ever present and has remained a daunting global challenge. Competing effectively in the dynamic marketplace is a major challenge for organisations, and with the Corona pandemic exerting unprecedent effects on organisations globally, most organisations are facing a more daunting challenge to survive (e.g. [65, 67]). Organisations must strive to strategically orientate their management accounting practices to enable them find ways to effectively navigate the daunting challenges they face in this Corona era.

### Implications of this study

The implications of this study are organised along managerial and theoretical implications.

*Managerial Implications*—Managers are reminded that optimal use of strategic management accounting techniques would boost organisational performance. Achieving high levels of organisational performance would however hinge on an organisation's ability to effectively align its SMA techniques usage to its internal and external dynamics. In other words, managers must bear in mind that there is no one-size fits all approach; therefore, they should endorse SMA techniques usage that fits their operational dynamics.

*Theoretical Implications*—In line with the central objective of this paper to sensitise the need for enhancing the understanding of the contingency perspective of strategic management accounting, the theoretical implications of this study are tailored towards specifying core gaps in the reviewed literature.

Overall, evidence from reviewed literature underlines the criticality of SMA techniques usage to organisational performance. Thus, if organisations strategically align SMA techniques usage to their operational setting, this would positively impact organisational performance. Within the goal of enhancing the literature on how to optimise the performance impact, much gaps still exist from the point off illuminating how differences in marketing and national culture differentiate SMA acceptance, usage, contingencies and performance impact.

Finally, on the point of performance, reviewed literature documents an obvious gap in the literature from the point of illuminating SMA techniques usage impact along the performance dimensions. As noted by Ojra [58], for some societies (especially ones that are Islamic cultured), non-financial performance is of central importance. Theoretical development from the point of SMA techniques usage, contingencies and non-financial performance impact is scanty.

### Limitations of the study

Based on systematic review approach, this study aimed to drive further knowledge development on the contingency perspective of strategic management accounting, drawing on the evidence from reviewed literature to understand the core debates in the literature and pinpoint directions for future research. Two core limitations of this research relate to the conceptual framework and volume of literature reviewed.

The conceptual framing of this study embraced only three themes in the SMA discourse, namely perceived environmental uncertainty, organisational structure and organisational strategy. Elaborated, the contingency premise considered in this study relates to perceived environmental uncertainty (competitive intensity, and market turbulence), organisational structure (formalisation and decentralisation), and organisational strategy. This study recognises that there are other contingencies of strategic management accounting practices that have not been included in the conceptual framing of this study.

To capture the central debates in the SMA discourse, extant literature was reviewed. It is however important to acknowledge that this study may have ignored some literature relevant to the conceptual premise of this study. Finally, although efforts were made by the researchers to ensure validity in this research by adopting an analytical approach that involved thorough reading of literature to ensure valid meaning in the interpretation, it must be reminded that subjectivity is a concern in every qualitative research.

### Future research directions

In explaining the theoretical implications of this study, core gaps in the literature were underlined (Section “Implications of this study”), while the limitations of this study were acknowledged in Section “Limitations of the study”. Building on these, this Section “Future research directions” extends the contribution of this study by specifying core directions for further knowledge development on the contingency perspective of strategic management accounting.

No doubt, this study has limitations, amongst which are the conceptual framework and the literature review scope. In their study, Naranjo-Gil et al. [54, p. 688] note that “future research is needed to examine other factors to add a more comprehensive view of management accounting”. Given the conceptual limitation of this study, this study reinforces the research call by Naranjo-Gil et al. [54]. Future research could expand the work done in this research and knowledge development by incorporating contingency factors that have not been considered in the conceptual framing of this study. More research is required in that regard, both from the point of a systematic literature review approach, as well as from the point of empirical investigations that seek to illuminate the contextual (industrial sectors and geographical settings) differentiators to the contingency impacts on the use of strategic management accounting techniques.

Furthermore, more research effort is required from the point of gaining deeper understanding of the various strategic management accounting techniques. Marketing dynamics (e.g. [62]) and national culture [35, 60] differ from one setting to another, therefore exploring the nature of strategic management accounting techniques that organisations endorse and why are core premises for research.

As flagged in the findings, there is a growing support of the notion that accounting practitioners do not subscribe to the use of the term strategic management accounting (e.g. CIMA^6^; [48, 55]). Further research could help to shed more light not only on why practitioners may not subscribe to the use of the term strategic management accounting, but also on the understanding of how practitioners would prefer to describe the management accounting practices that they embrace, and also why the specific practices are prioritised.

Furthermore, on the point of the content of strategic management accounting, researchers have also noted that not much effort is given to highlighting clearly the accounting information that organisations should give much attention to towards boosting organisational performance (e.g. [53, 89]). Future research should aim to fill this gap. Doing that is critical to fully optimising the performance benefits of strategic management accounting [56].

Reviewed literature has documented that the extent to which strategic management accounting practices would aid management decision-making and organisational performance would depend on the contingency dynamics of the organisation (e.g. [11, 58]). Understanding the contingency premise of strategic management accounting utility in driving effective management decision-making and organisational performance is a critical research premise, and future research should aim to shed more light on that. No one size fits all approach that works for all organisations in all contexts. Therefore, future research should seek to enhance the 'fit' foundation of strategic management accounting relevance and performance outcome. In that regard, future research should seek to illuminate further how perceived environmental uncertainty, decentralisation, formalisation, strategy and other contingency factors not considered in this study, would influence strategic management accounting techniques usage and organisational performance impact. In the particular case of perceived environmental uncertainty, more research is not only required from the point of understanding the influence of the construct, but also clarifying the competitive intensity and market turbulence associations.

An insight that emerged from the reviewed literature relates to the fact that majority of efforts to improve strategic management accounting discourse have considered mainly financial aspects of organisational performance (e.g. [58, 86]). Focusing only on financial performance is inadequate as the customer perspective of performance is neglected [45, 58]. The importance of focusing on customer performance has been re-echoed in further literature: organisational-level customer satisfaction associates positively to financial performance (e.g. [24, 86]), and customer performance enables business strategy and an organisation's ability to deliver value to its shareholders as well as customers [25]. Supporting prior research (e.g. [49, 58, 86]), this study underlines the need for more studies that illuminate non-financial performance aspects and strategic management accounting association.

Finally, the Corona pandemic, which remains a global crisis, has exerted unprecedent global economic damage. Organisations are facing daunting challenges as a result of the Corona pandemic and are still seeking ways to successfully navigate these challenges. Future research should illuminate what strategic management accounting practices organisations are endorsing in the effort to effectively navigate the Corona-crisis-induced challenges.

## Data Availability

This study is based on the review of literature.

## Abbreviations

SMA: Strategic management accounting
SMAU: Strategic management accounting usage
CIMA: Chartered Institute of Management Accountants
MACS: Management accounting and control system
MCS: Management control systems
QMS: Quality management system

## Footnotes

1 Management Accounting in support of the strategic management process. https://www.cimaglobal.com/Documents/Thought_leadership_docs/Management%20and%20financial%20accounting/Academic-Research-Report-Strategic-Management-Process.pdf.

2 Management Accounting in support of the strategic management process. https://www.cimaglobal.com/Documents/Thought_leadership_docs/Management%20and%20financial%20accounting/Academic-Research-Report-Strategic-Management-Process.pdf.

3 Management Accounting in support of the strategic management process. https://www.cimaglobal.com/Documents/Thought_leadership_docs/Management%20and%20financial%20accounting/Academic-Research-Report-Strategic-Management-Process.pdf.

4 Management Accounting in support of the strategic management process. https://www.cimaglobal.com/Documents/Thought_leadership_docs/Management%20and%20financial%20accounting/Academic-Research-Report-Strategic-Management-Process.pdf.

5 January 9—WHO Announces Mysterious Coronavirus-Related Pneumonia in Wuhan, China.

6 Management Accounting in support of the strategic management process. https://www.cimaglobal.com/Documents/Thought_leadership_docs/Management%20and%20financial%20accounting/Academic-Research-Report-Strategic-Management-Process.pdf.
